# Artochalcone—A Geranylated Dihydrochalcone from Male Inflorescences of *Artocarpus altilis* (Breadfruit) with Potent Anti-Tyrosinase Activity

**DOI:** 10.3390/molecules31162752

**Published:** 2026-08-07

**Authors:** Kenna L. Whitnell, Jackson J. Villemaire-McCutcheon, Ulli K. C. Bodnar, M. Sameer Al-Abdul-Wahid, Tariq A. Akhtar

**Affiliations:** 1 Department of Molecular and Cellular Biology, University of Guelph, Guelph, ON N1G 2W1, Canada; kennawhitnell@gmail.com (K.L.W.); jvillema@uoguelph.ca (J.J.V.-M.); ubodnar@uoguelph.ca (U.K.C.B.); 2 NMR Centre, University of Guelph, Guelph, ON N1G 2W1, Canada; s.wahid@uoguelph.ca

**Keywords:** *Artocarpus altilis*, breadfruit, anti-tyrosinase, skin-whitening agent, antioxidant

## Abstract

A key factor that regulates melanogenesis is the activity of tyrosinase, and inhibitors of this enzyme are used as skin-whitening and lightening agents in the skincare and cosmetics industries. The methanolic extract from male inflorescences of *Artocarpus altilis* (Parkinson) Fosberg (breadfruit) was investigated for its bioactivities which are important for the skincare industry, including anti-tyrosinase activity. Bioassay-guided fractionation led to the isolation of a geranylated dihydrochalcone, 2-Geranyl-2’,3,4,4’-tetrahydroxydihydrochalcone (hereinafter referred to as Artochalcone), present at 1.64% of the inflorescence dry weight. Its structure was determined by a combination of 1D and 2D-NMR and HR-ESI-MS analysis. Artochalcone was found to inhibit tyrosinase with an IC_50_ value of 28.4 μM, making it sixteen times more effective than the leading commercial skin-whitening agent, arbutin. In addition, Artochalcone displayed significant antioxidant activity determined by 2,2-diphenyl-1-picrylhydrazyl (IC_50_ = 20.4 μM) and nitric oxide scavenging assays (IC_50_ = 20.7 μM).

## 1. Introduction

*Artocarpus altilis* (breadfruit) is a tropical tree that is cultivated in Pacific island agriculture for its highly nutritious fruit [1]. Breadfruit originated in New Guinea, but it is now widely found throughout Southeast Asia and was brought to the Caribbean as a cheap food source for sugar plantation labourers in the 1700s. In addition to its utility as a staple food, fibres from the bark of the breadfruit tree are used in clothing, crafts and fabrics, and other parts of the plant have medicinal properties that have benefited Pacific Islanders for thousands of years [2]. For example, latex from the breadfruit tree has been used topically to treat broken bones, sprains and infections, and various skin ailments and fungal diseases have been treated with crushed leaves and/or heartwood of the tree [3,4]. Finally, the burning of sun-dried clusters of breadfruit male inflorescences is a widely practiced folk remedy to repel flying insects [5].

Many of the health-promoting properties of breadfruit appear to be linked to the presence of flavonoids that accumulate within the various organs of the plant [6,7,8,9,10]. In addition to the anti-inflammatory, anti-cancer and antioxidant activities attributed to these compounds, inhibition of tyrosinase enzyme activity has also been reported [11,12,13,14,15,16]. Since tyrosinase controls the production of melanin in skin melanocytes, agents that function as inhibitors of the enzyme offer protection against UV-induced melanogenesis and produce skin-whitening effects—two highly valued qualities in the cosmetics and skincare industries [16,17,18]. Notably, geranylated flavones have previously been recovered from dried Artocarpus bud covers, indicating that reproductive tissues accumulate these specialized metabolites [19]. Moreover, male inflorescences are generally non-food tissues that can be harvested without compromising breadfruit production, offering a potentially sustainable source of high-value natural cosmeceutical compounds. Accordingly, in this study, a methanolic extract of breadfruit male inflorescences (flowers) was fractionated in order to identify specific flavonoids that exhibit potent anti-tyrosinase activity. This analysis resulted in the identification of 2-geranyl-2’,3,4,4’-tetrahydroxydihydrochalcone, a prenylated flavonoid which is hereinafter referred to as Artochalcone.

## 2. Results and Discussion

**Figure 1:**
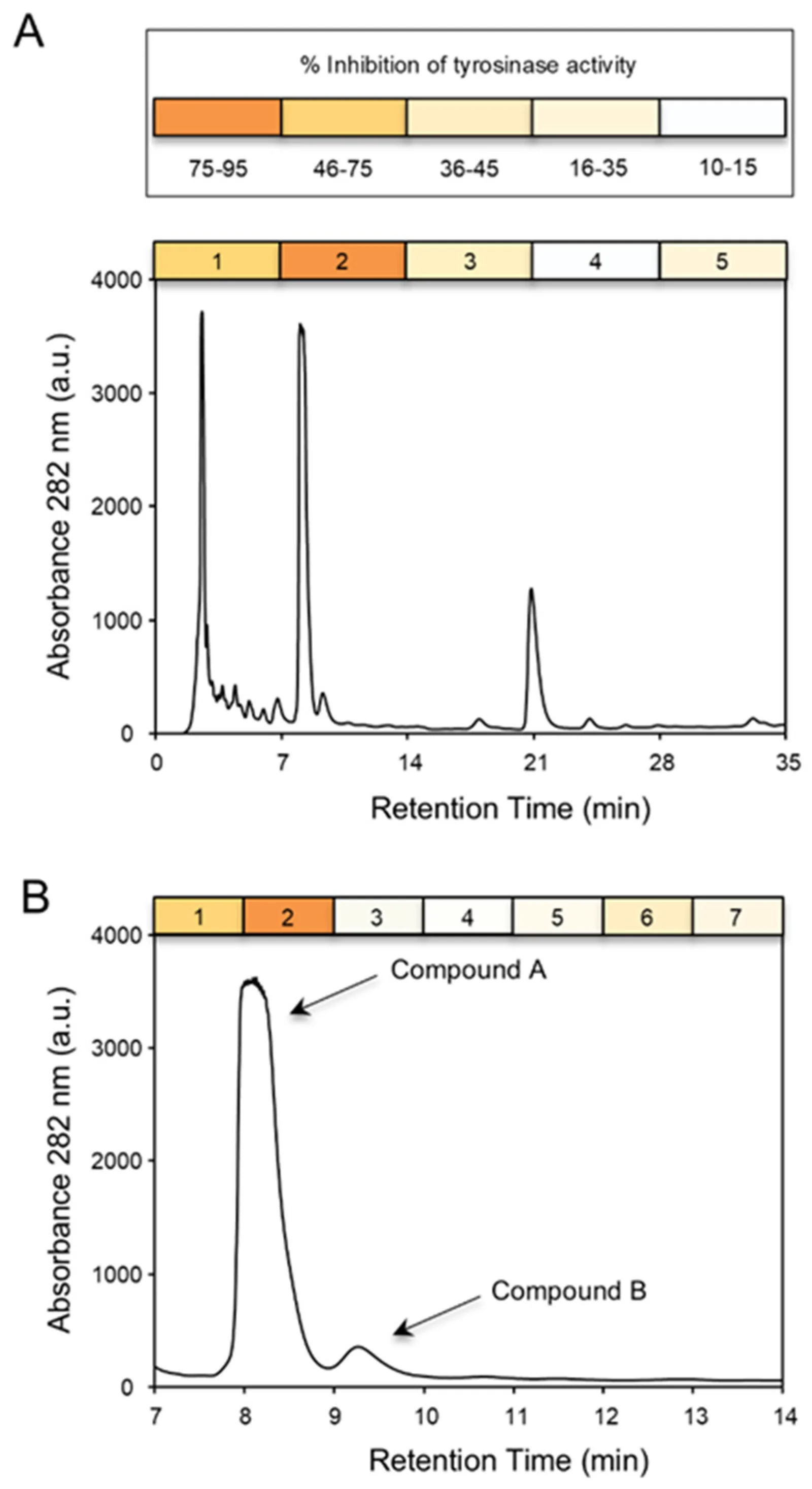
Assay-guided fractionation of breadfruit flower extracts. (**A**) Methanolic extracts of male breadfruit flowers were separated by reversed-phase HPLC, and fractions were collected every seven minutes to obtain five initial fractions (numbered 1 through 5) containing compounds that absorbed strongly at 282 nm. Each fraction was tested for anti-tyrosinase activity and scored for their percent inhibition (see accompanying legend above). Note that the second fraction (‘fraction 2’, collected between 7 and 14 min) exhibited the most potent inhibition of tyrosinase. (**B**) Further separation of anti-tyrosinase activity. ‘Fraction 2’ contained two UV-absorbing compounds (compound A and compound B) and was re-chromatographed as shown above to obtain seven additional fractions (numbered 1 through 7) that were scored for anti-tyrosinase activity.

**Figure 2:**
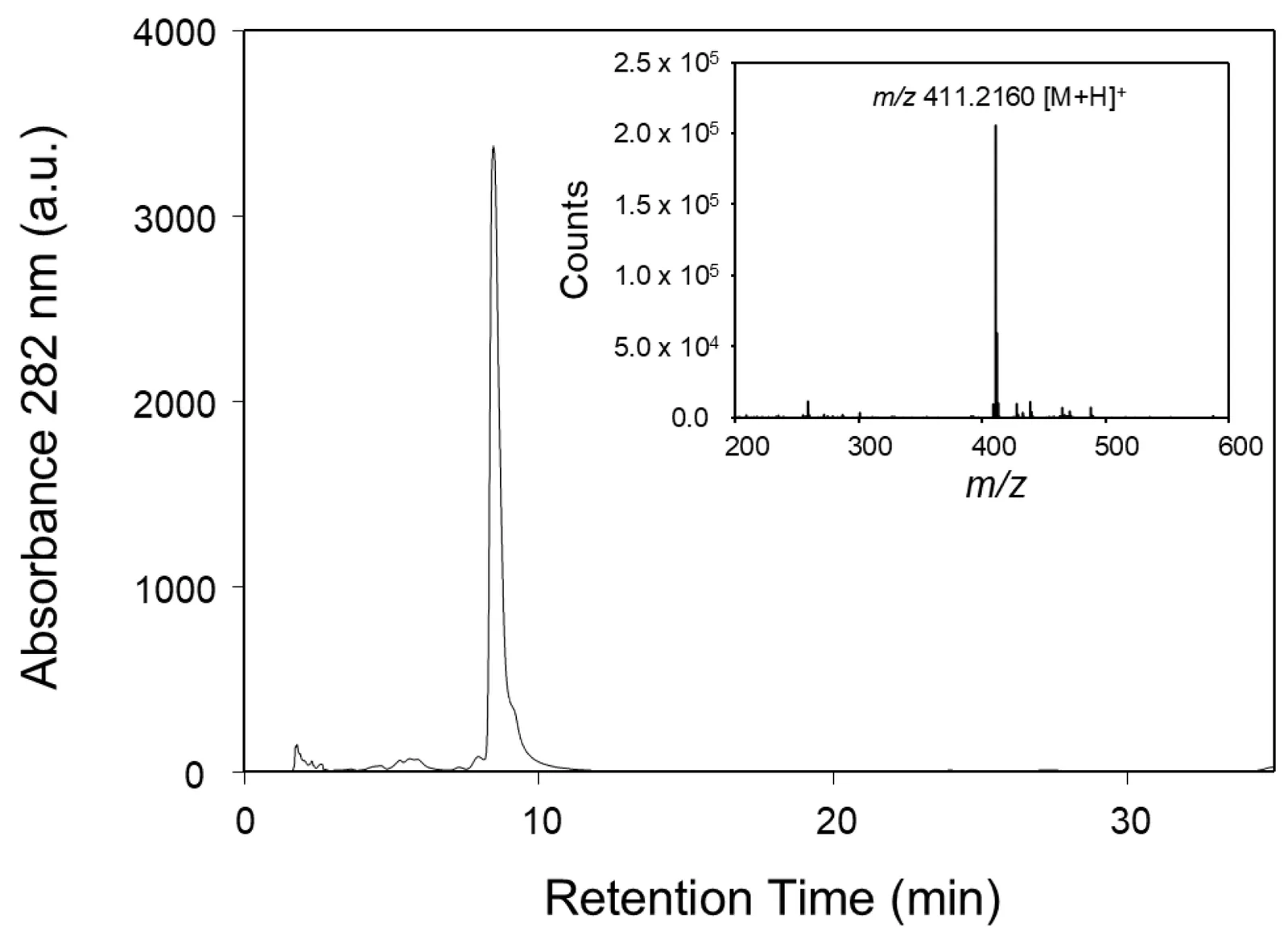
Purification and HR-ESI-MS characterization of a potent anti-tyrosinase inhibitor from breadfruit. Assay-guided fractionation of *Artocarpus altilis* (breadfruit) male flower extracts led to the identification of a UV-absorbing metabolite (compound A), which was subsequently purified by HPLC as a yellow oil. High-resolution electrospray ionization mass spectrometry (HR-ESI-MS; inset) established the molecular formula C_25_H_30_O_5_, with an observed ion at *m*/*z* 411.2160 ([M+H]^+^), in agreement with the calculated value for C_25_H_31_O_5_^+^ (*m*/*z* 411.2161; neutral formula C_25_H_30_O_5_), corresponding to a mass error of approximately 0.2 ppm. The HR-ESI-MS data provide the principal mass spectrometric evidence supporting the proposed molecular formula.

A methanolic extract of breadfruit flowers was first evaluated for its anti-tyrosinase activity and shown to exhibit an IC_50_ value of 9.6 µg mL^−1^. Strikingly, this inhibitory activity is apparently ten times more potent than previously reported anti-tyrosinase activities within various plant extracts that are considered rich sources of tyrosinase inhibitors [20,21,22,23]. To identify the causal inhibitory agent(s) within the breadfruit inflorescences, the flower extract was subjected to reversed-phase high-performance liquid chromatography to obtain five fractions (Figure 1A). Among these, fraction 2 potently inhibited tyrosinase enzyme activity and was composed of two major components (compound A and B) that absorbed strongly at 282 nm (Figure 1B). The anti-tyrosinase activity within this fraction was further partitioned into seven additional fractions and it was determined that virtually all the inhibitory activity was captured in a single fraction (fraction 2), which interestingly contained compound A. This UV-absorbing compound was subsequently isolated as a yellow oil and the molecular formula was determined as C_25_H_30_O_5_ based on HR-ESI-MS analysis (*m*/*z* [M+H]^+^ 411.2160, calcd. for C_25_H_30_O_5_^+^, 411.2166; Figure 2).

Next, compound A was subjected to structural analysis (Table 1), and based on the observed chemical shifts, integrations, and J-couplings in the ^1^H and COSY NMR spectra, each of the ^1^H peaks (and their associated ^13^C shifts) was assigned to one of four spin systems: a geranyl moiety; a 1,2,4-trisubstituted benzene ring; a 1,2,3,4-tetrasubstituted benzene ring; and an ethyl group. Tentative COSY assignments for the geranyl methyl groups (δ_C_ 24.4 C-23, δ_C_ 16.3 C-24, and δ_C_ 15.0 C-25) were confirmed by HMBC correlations to protonated vinyl carbons (δ_C_ 123.7 C-17 and δ_C_ 123.9 C-21) and unprotonated vinyl carbons (δ_C_ 133.9 C-18 and δ_C_ 130.7 C-22), thus completing assignment of the geranyl group. Protons H-3, H-5, and H-6 were conclusively assigned based on the well-defined J-coupling patterns around a 1,2,4-trisubstituted benzene ring, and HMBC correlations from these three protons were used to assign unprotonated ring carbons (δ_C_ 112.5 C-1, δ_C_ 165.0 C-2, and δ_C_ 165.0 C-4) by way of the intense ^3^J_CH_ couplings in aromatic ring systems, providing full assignment of the trisubstituted ring. An HMBC correlation from trisubstituted ring proton H-6 to a carbon at 204.4 ppm was assigned as a carbonyl (δ_C_ 204.4 C-7) attached to the ring at the C-1 position. This carbonyl, in turn, was determined to be attached to the ethyl group by way of two HMBC peaks from the ethyl group to carbonyl C-7: a stronger correlation from the 3.08 ppm CH_2_ protons (assigned as H-8) and a weaker correlation from the 2.87 ppm CH_2_ protons (assigned as H-9). The NMR spectra are provided in the Supplementary Materials (Figures S1–S6).

**Figure 3:**
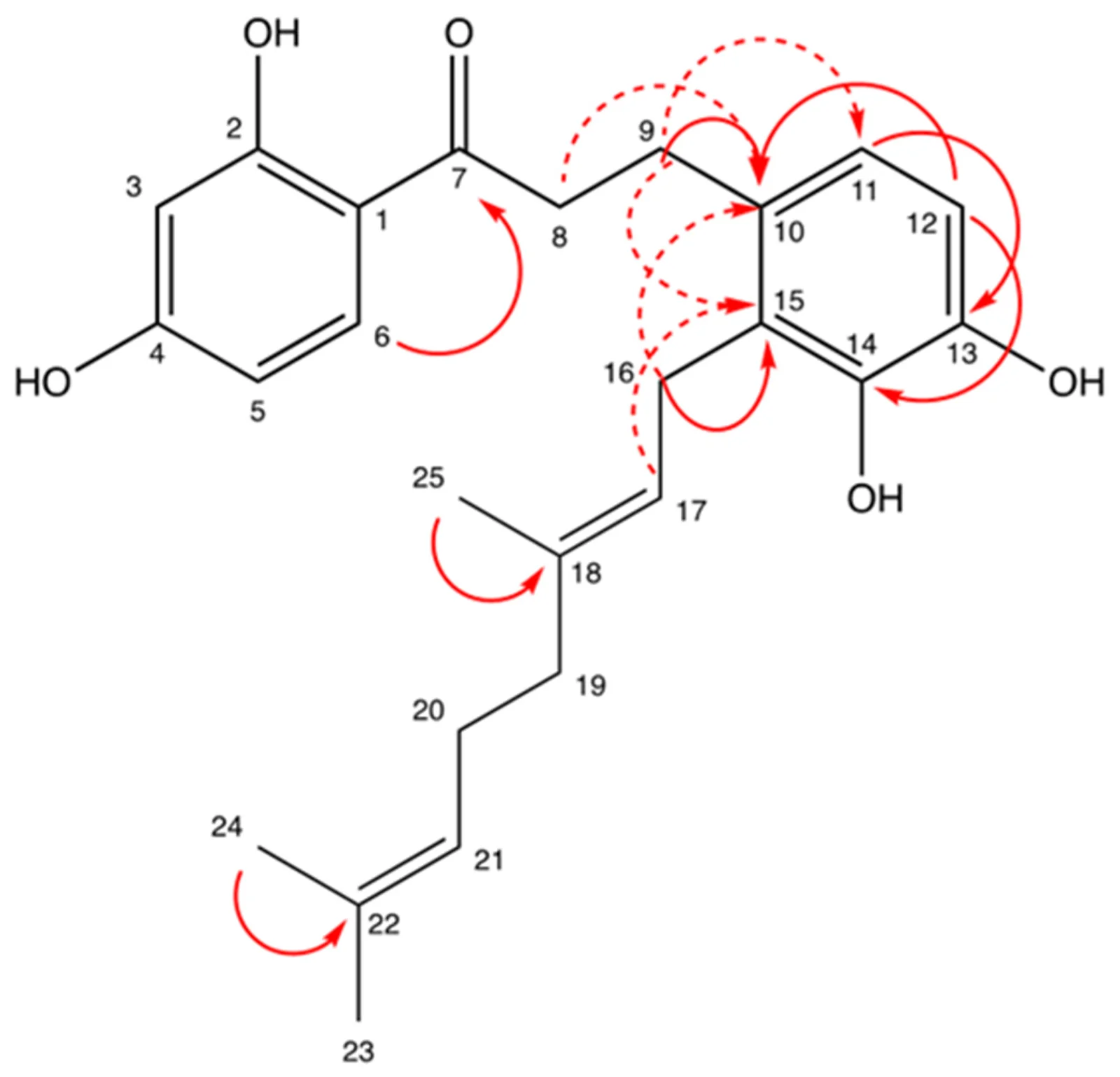
Key HMBC correlations for (E)-1-(2,4-dihydroxyphenyl)-3-(2-(3,7-dimethylocta-2,6-dien-1-yl)-3,4-dihydroxyphenyl)propan-1-one. Arrows indicate magnetization transfer from a proton to a carbon; dashed arrows indicate less intense (i.e., longer range) HMBC peaks.

In regard to the tetrasubstituted ring, intense ^3^J_CH_ HMBC crosspeaks from the two *ortho* protons, H-11 and H-12, provided the chemical shifts in the four unprotonated ring carbons (C-10, C-13, C-14, and C-15), but not their conclusive assignments. Three HMBC correlations from H-9 ethyl group protons and two HMBC correlations from geranyl group protons (a weak putative ^3^J_CH_ from H-17 and a stronger putative ^2^J_CH_ from H-16) to these four unprotonated ring carbons were instrumental in completing the assignment (Figure 3). A weak COSY between H-9 and H-11 further supports the determined structure.

The final remaining question is that of the unknown substituents on four aromatic carbons (two on each ring). All four carbons have a chemical shift of over 140 ppm (suggesting an electronegative substituent), and the molecular formula obtained by mass spectrometry contains four oxygen and four hydrogen atoms not previously accounted for in the NMR assignments, indicating that these four substituents must be OH groups. Collectively, this analysis permitted identification of compound A as 2-geranyl-2’,3,4,4’-tetrahydroxydihydrochalcone, hereinafter referred to as Artochalcone. It should be noted that this compound has been previously identified in breadfruit and shown to act as an antimalarial agent [24], an antioxidant [25], and an inhibitor of cathepsin K, a cysteine protease that is involved osteoporosis [19]. In light of previous studies demonstrating that dihydrochalcones can inhibit tyrosinase [26], we remained interested in evaluating the potential of Artochalcone as an inhibitor of melanogenesis.

Artochalcone was next evaluated for its specific inhibition of tyrosinase activity, alongside arbutin, a well-known tyrosinase inhibitor and leading skin-whitening agent used in the cosmetics and skincare industries [27,28]. Our assay examined the ability of Artochalcone to inhibit the first catalytic step of tyrosinase—conversion of tyrosine to dihydroxyphenylalanine. Inhibition of this step is especially important as the second catalytic step can occur spontaneously under physiological conditions [28], meaning sole enzymatic inhibition would be sufficient to prevent pigmentation. Consistent with previous reports, it was determined that arbutin inhibited tyrosinase activity with an IC_50_ value of 471.5 µM [8]. Interestingly, it was observed that the initial methanolic extract was a more potent tyrosinase inhibitor, leading us to speculate that there are likely additional metabolites in this tissue that inhibit tyrosinase activity or act in synergy with Artochalcone. Comparatively, Artochalcone exhibited anti-tyrosinase activity that was approximately sixteen times more effective, with an IC_50_ value of 28.4 µM (Table 2). The Artochalcone content in dried breadfruit flowers was relatively high at 1.6% (*w*/*w*), compared to 0.0002657% (*w*/*w*) in the dried leaf tissue [28]. Breadfruit flowers therefore have the potential to provide the skincare and cosmetics industries with a novel and rich source of anti-tyrosinase agents. While tyrosinase inhibition is widely regarded as a key indicator of antimelanogenic potential, the present findings are based solely on an in vitro enzyme assay. Future studies evaluating melanin production in melanocytes and skin irritation in relevant biological models will therefore be necessary to confirm the cosmetic applicability of Artochalcone.

**Figure 4:**
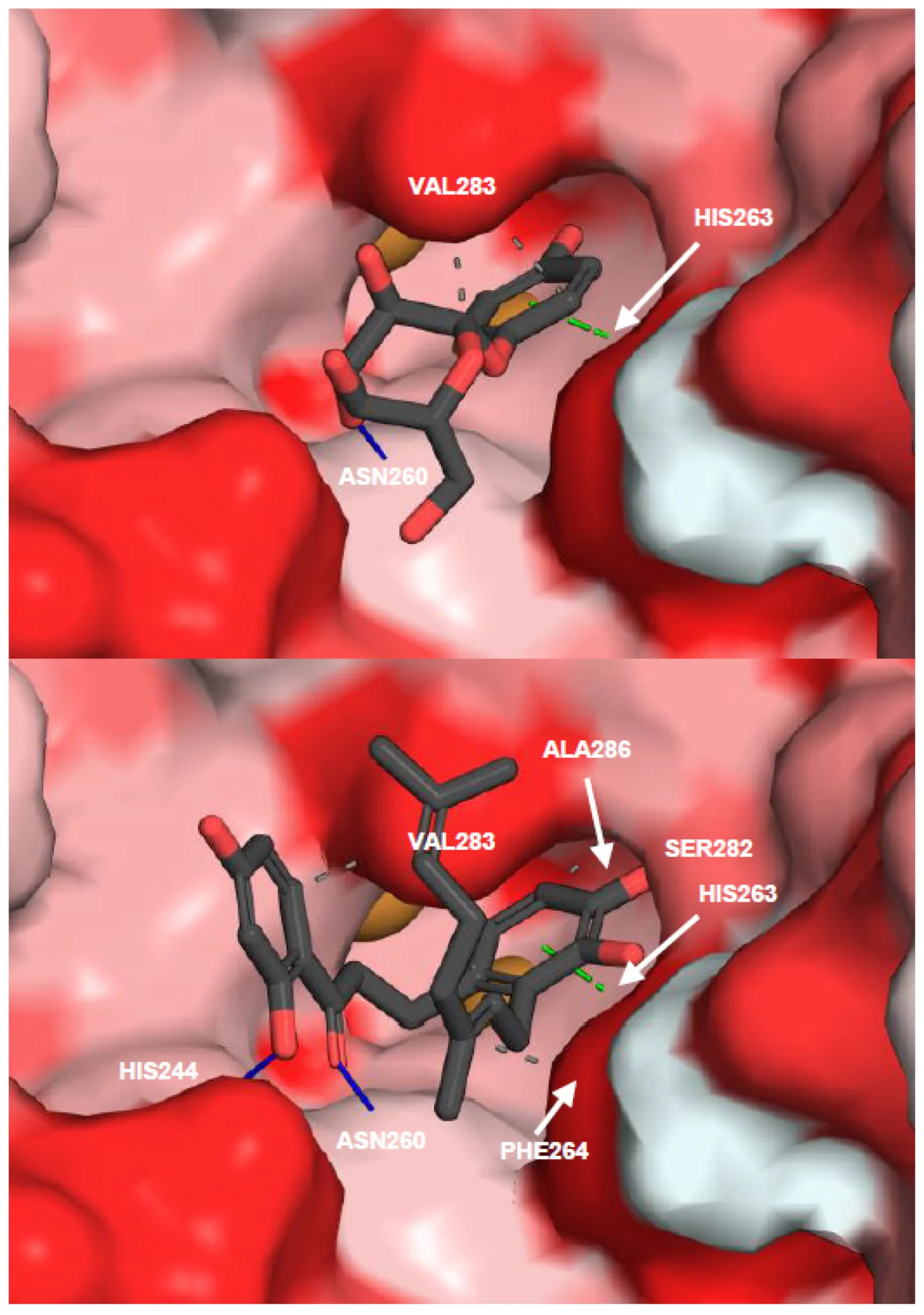
In silico docking poses of arbutin (**top**) and artochalcone (**bottom**) within the tyrosinase active site. The protein surface is shown coloured according to the Eisenberg hydrophobicity scale (red = greater hydrophobicity). Copper ions are shown in orange. Ligands are coloured by atom type (C = grey; O = red; and H = white). Protein–ligand interactions are depicted by lines and are coloured by type (hydrophobic interaction = grey; hydrogen bond = blue; and π-stacking = green). Refer to the online version of this article for colour interpretation.

Consistent with the anti-tyrosinase assay results, molecular docking simulations revealed that arbutin exhibited a binding affinity of −5.817 kcal/mol, whereas non-prenylated Artochalcone displayed a stronger affinity (−7.096 kcal/mol). Prenylated Artochalcone showed the highest binding affinity (−7.454 kcal/mol), supporting its superior inhibitory activity against tyrosinase. Flavonoids have been shown to inhibit tyrosinase both competitively and noncompetitively [28], sometimes by targeting and chelating the binuclear copper ions present in the active site [29]. While the flavonoid scaffold can confer potent tyrosinase inhibitory potential [30], interaction profiling revealed that prenylated Artochalcone maintained three hydrogen bonds and a parallel π–π stacking interaction while exhibiting a greater number of hydrophobic interactions than the non-prenylated analogue, particularly involving Val283 and Ala286 (Table 3). The dominant predicted binding affinity of prenylated Artochalcone may be associated with the presence of the geranyl moiety, where it might plausibly interact with hydrophobic residues adjacent to the receptor’s active site. As observed in Figure 4, this association may stabilize the flavonoid in an orientation that positions it close to the binuclear copper centre, potentially supporting previously reported copper-chelating and competitive inhibition mechanisms of flavonoids. This observation may also account for the substantially higher IC_50_ values reported for non-prenylated dihydrochalcones compared with Artochalcone [24,26]. However, this proposed mechanism remains hypothetical and was not definitively demonstrated by the docking analysis.

Oxidative stress is a major contributor to skin ageing, inflammation, and UV-induced hyperpigmentation, making antioxidant activity a highly desirable property of natural ingredients intended for cosmetic and skincare applications. Accordingly, the antioxidant potential of Artochalcone was evaluated using the complementary DPPH radical and nitric oxide scavenging assays, two well-established methods for assessing the free-radical scavenging capacity of plant-derived polyphenols and their ability to neutralize biologically relevant reactive nitrogen species [31,32]. Artochalcone was first evaluated for antioxidant potential by monitoring its radical scavenging activity against 2,2-diphenyl-1-picrylhydrazyl (DPPH). Artochalcone readily converted the DPPH radical to its reduced hydrazine derivative with an IC_50_ value of 20.4 µM (Table 2), which was comparable to the radical scavenging activity of quercetin (IC_50_ = 7.5 μM), a ubiquitous dietary flavonol with well-established antioxidant activity [8,31]. This reducing ability of artochalcone is likely in part due to the catechol substructure found on its B ring, as this moiety is known to markedly increase the free-radical scavenging ability of polyphenols [32]. The strong antioxidant activity of Artochalcone offers yet another merit for its use in a variety of skincare and cosmetic applications, as reactive oxygen species (ROS) produced upon absorption of UV radiation induce the release of α-melanocyte-stimulating hormone, which increases melanogenesis and can lead to skin hyperpigmentation [33].

The UV-induced production of ROS at the skin surface also activates the inducible nitric oxide synthase, leading to overproduction of nitric oxide (NO) and potential inflammatory skin conditions [34,35]. Therefore, the NO scavenging activity of Artochalcone was next tested and compared to that of ascorbic acid, a standard for the estimation of NO scavenging activity [35]. Artochalcone proved to be an extremely effective NO scavenger with an IC_50_ value of 20.7 μM—approximately 120 times more potent than ascorbic acid (Table 2). Taken together, the ability of Artochalcone to reduce NO to the less reactive nitrite, coupled with its favourable anti-tyrosinase and antioxidant properties, therefore offers a suit of desirable properties in a variety of topical skincare applications for this compound.

## 3. Materials and Methods

### 3.1. General Methods

Arbutin, mushroom tyrosinase, quercetin, tyrosine, sodium nitroprusside, 2,2-diphenyl-1-picrylhydrazyl, and the Griess Reagent were purchased from Sigma-Aldrich (Oakville, ON, Canada). All other reagents that were used in this study were purchased from Thermo-Fisher Scientific (Ottawa, ON, Canada). A Multiscan Go 96-well plate reader running SkanIt Software 4.1 (Thermo-Fischer Scientific) was utilized for all in vitro assays.

### 3.2. Plant Material

The male inflorescences of *A. altilis* (Parkinson) Fosberg (breadfruit) were collected in Apia, Samoa (Open Location Code: 569G+4CP), and were sun-dried for approximately two weeks. Any inflorescences that did not dry properly and/or were infected with mould were discarded. The inflorescences were packaged into plastic bags, boxed, and shipped to Guelph, ON, Canada, for research purposes. The plant material was identified and authenticated by Dr. Carole Ann Lacroix and a voucher specimen (No. 102032) was deposited at the Ontario Agricultural College Herbarium in Guelph, ON, Canada.

### 3.3. Fractionation of Breadfruit Extracts and Purification of Artochalcone

The dried flowers (10 g) were extracted in methanol using a soxhlet apparatus for 8 h at 70 °C, with 0.1 g mL^−1^ of dry plant material. After extraction, the breadfruit flower extract was stored at 4 °C for 24 h to facilitate fatty acid precipitation and then concentrated to 20 mL using a rotary evaporator. Concentrated extracts were next fractioned using an Agilent 1260 Infinity HPLC system equipped with a Spherisorb S5ODS2 reverse-phase column (250 mm × 4.6 mm, 5 μm; Supelco, Oakville, ON, Canada). Compounds (detected by absorbance at 282 nm) were eluted from the column at a flow rate of 1 mL min^−1^ according to the following method: 10 min with 80% methanol, followed by a 10 min of linear gradient from 80 to 90% MeOH, and a final isocratic washing step for 5 min with 90% MeOH. Initially, five fractions were obtained from this crude extract by manually collecting the eluate every seven minutes. Each fraction was subsequently evaluated for their anti-tyrosinase activity (see below), and the second fraction showed the highest bioactivity. This fraction was further separated using the above method by collecting the eluate every one minute to obtain seven fractions that were then assayed accordingly. This led to the isolation of one highly bioactive fraction (‘fraction 2’) that consisted of a single UV-absorbing compound at 282 nm. The compound was collected manually and re-chromatographed to ensure purity before structural characterization. A standard curve of apigenin was used to determine the approximate mass of unknown breadfruit flavonoids detected by the HPLC. Further details of the assay-guided purification procedure are provided in Figure 1.

### 3.4. HR-ESI-MS

High-resolution LC–MS analyses were carried out using an Agilent 1200 HPLC system coupled to an Agilent UHD 6530 quadrupole time-of-flight (Q-TOF) mass spectrometer. Samples were introduced directly into the mass spectrometer without chromatographic separation; however, a C18 guard cartridge (2.1 mm i.d., 5 μm; Agilent Technologies, Mississauga, ON, Canada) was installed upstream of the ion source to protect the injection line. The mobile phase consisted of 0.1% formic acid in a 50:50 (*v*/*v*) acetonitrile/water mixture delivered at 0.4 mL min^−1^. Electrospray ionization was performed using a capillary voltage of 4.0 kV, a drying gas temperature of 250 °C, and a drying gas flow of 8 L min^−1^. The nebulizer pressure was maintained at 30 psi, while the fragmentor, nozzle, skimmer, and octapole RF voltages were set to 160, 1000, 65, and 750 V, respectively. Nitrogen (purity > 98%) served as the nebulizing, drying, and collision gas. Mass spectra were acquired in positive ion mode over an *m*/*z* range of 50–1500 using the 4 GHz extended dynamic range setting. Data were collected using either targeted MS/MS or data-independent acquisition at an MS/MS scan rate of 1.41 spectra s^−1^, with an overall acquisition rate of two spectra per second. Prior to analysis, mass calibration was performed using Agilent tuning mix HP0321 (Agilent Technologies) prepared in acetonitrile. The analytical confirmation of the isolated Artochalcone by LC-HR-ESI-MS is presented in Figure S1.

### 3.5. NMR Assignment

(*E*)-1-(2,4-dihydroxyphenyl)-3-(2-(3,7-dimethylocta-2,6-dien-1-yl)-3,4-dihydroxyphenyl)propan-1-one: ^1^H NMR (Methanol-d4, 600 MHz): δH 7.63 (d, *J* = 8.9, H–C(6)), 6.58 (d, *J* = 8.1, H–C(12)), 6.53 (d, *J* = 8.1, H–C(11)), 6.31 (dd, *J* = 8.8, 2.4, H–C(5)), 6.25 (d, *J* = 2.4, H–C(3)), 5.13 (bt, *J* = 6.6, H–C(17)), 5.03 (bt, *J* = 7.0, H–C(21)), 3.39 (bd, *J* = 6.5, H_2_–C(16)), 3.07–3.10 (m, H_2_–C(8)), 2.86–2.89 (m, H_2_–C(9)), 2.03 (q, *J* = 7.2, H_2_–C(20)), 1.94 (t, *J* = 7.6, H_2_–C(19)), 1.72 (s, H_3_–C(25)), 1.59 (s, H_3_–C(23)), 1.52 (s, H_3_–C(24)); ^13^C NMR (Methanol-d4, 150 MHz)): δC 204.4 (C(7)), 165.0 (C(4)), 165.0 (C(2)), 143.1 (C(14)), 142.9 (C(13)), 133.9 (C(18)), 132.3 (C(6)), 130.9 (C(10)), 130.7 (C(22)), 126.6 (C(15)), 123.9 (C(21)), 123.7 (C(17)), 119.6 (C(11)), 112.5 (C(1)), 112.2 (C(12)), 107.7 (C(5)), 102.3 (C(3)), 39.4 (C(8)), 39.4 (C(19)), 27.5 (C(9)), 26.3 (C(20)), 24.7 (C(16)), 24.4 (C(23)), 16.3 (C(24)), 15.0 (C(25)). Further details of NMR characterization are presented in Figures S2–S6.

### 3.6. Antioxidant Assay

Assays to assess antioxidant potential were performed using the 2,2-diphenyl-1-picrylhydrazyl (DPPH) radical scavenging assay as described in [36] with minor modifications. Stock solutions of DPPH (0.039 mg mL^−1^), test compound (50 mM) or the quercetin positive control (50 mM) were prepared in methanol and stored at 4 °C. To each well of a 96-well microtiter plate, 100 µL of DPPH solution and 100 µL of various concentrations of test compound (1–100 µM) or positive control (1–40 µM) were then added. Absorbance readings at 520 nm were taken at 60 s intervals for 30 min at room temperature. Percentage of total radical scavenging activity is calculated based on the following formula:

$$Percentage\;of\;total\;radical\;scavenging\;activity=\frac{\left({OD}_{control}-{OD}_{sample}\right)}{{OD}_{control}}\times 100\%$$

### 3.7. Nitric Oxide Scavenging Assay

Assays to assess nitric oxide (NO) scavenging activity were performed according to previous methods [37]. Briefly, a 10 mM solution of sodium nitroprusside (SNP) was incubated alone or in the presence of various concentrations of test compound (1–100 µM) or with ascorbic acid (2–8 mM) as a positive control. Incubations occurred for 60 min at room temperature under ambient light and the NO scavenging activity was determined by measuring nitrite formation in the SNP solutions according to the Griess assay [38]. Equal volumes of Griess Reagent (1% sulfanilamide and 0.1% naphtylethyenediamine dihydrochloride in 2.5% H_3_PO_4_) and SNP solutions were incubated together at room temperature. The optical density was measured at 550 nm using a microplate reader and NO scavenging activity was calculated by the following formula:

$$Percentage\;of\;total\;NO\;scavenging\;activity=\frac{\left({OD}_{control}-{OD}_{sample}\right)}{{OD}_{control}}\times 100\%$$

### 3.8. Anti-Tyrosinase Assay

Assays to assess anti-tyrosinase activity were performed as previously described [39], with minor modifications. A 70 µL solution of 50 mM potassium phosphate containing 30 μL of mushroom tyrosinase (333 U mL^−1^) were added into the wells of a 96-well microtiter plate and 2 µL of various test compound solutions (1–5 mM) or arbutin (25–200 mM), a well characterized tyrosinase inhibitor, were then added into each well. After five minutes of incubation at room temperature, 100 μL of L-Tyrosine (2 mM) was added to each well and absorbance was then measured at 492 nm immediately and after 30 min of further incubation. Anti-tyrosinase activity was calculated according to the following formula:

$$\begin{array}{c} Percentage\;of\;anti-tyrosinase\;activity\\ \;=\frac{\left({OD}_{control\;t=30\min}-{OD}_{control\;t=0\min}\right)-\left({OD}_{sample\;t=30\min}-{OD}_{sample\;t=0\min}\right)}{\left({OD}_{control\;t=30\min}-{OD}_{control\;t=0\min}\right)}\times 100\% \end{array}$$

### 3.9. Molecular Docking Simulations

Molecular docking simulations were conducted based on the workflow described by Sadeghpour et al. (2026) [40]. The 3D crystal structure of A. bisporus tyrosinase (PDB accession code: 2Y9X) was obtained from the RCSB Protein Data Bank (RCSB Protein Data 2013) (https://www.rcsb.org/). Using ProDy v2.5.0 as a selection tool, the binuclear copper ions within the active site were retained while all other non-protein atoms were removed from the structure. The CRYST1 record was preserved, and the receptor was protonated using the reduce package. The receptor was then prepared and saved in PDBQT format using prepare receptor from the ADFR suite. All ligands were prepared using the same workflow. By supplying Molscrub with the SMILES strings corresponding to each ligand, protonated (pH 7.4) 3D structures were generated and saved in SDF format. Ligands were then prepared for docking using mk_prepare_ligand from Meeko and saved in PDBQT format. The docking grid was centred at x = −9.674, y = −26.845, z = −41.239, with the grid centre defined as the centre of mass of the active site residues defined by Ismaya et al. (2011) [41], the binuclear copper atoms, and the co-crystalized inhibitor, tropolone. The grid dimensions were set to 24 × 24 × 24 Å to fully encompass the binding pocket and accommodate the relatively large ligands. Molecular docking simulations were performed using AutoDock Vina v1.2.6 with an exhaustiveness of 500 [42,43]. Ten docking poses were generated for each ligand, and the pose with the lowest predicted free energy of binding was chosen for visualization and prepared for publication using PyMol (3.1.3.1) [44]. Protein–ligand interactions were identified using the Protein–Ligand Interaction Profiler (PLIP) [45]. The receptor surface was coloured according to the Eisenberg hydrophobicity scale [46].

### 3.10. Data Analysis

Data was analyzed using Microsoft Excel. All data points used for analysis are the mean ± standard deviation of three independent measurements. IC_50_ values were determined by fitting dose–response curves using nonlinear regression, and the reported values represent the mean ± standard deviation of three independent experiments. NMR data was analyzed using Bruker TopSpin (v3.6.5), and, ass spectrometer data acquisition and analysis were performed with MassHunter^®^ Workstation software (B.04.00).

## 4. Conclusions

Collectively, these findings establish Artochalcone as a promising lead molecule for the development of next-generation natural skin-lightening agents and demonstrate that breadfruit male inflorescences represent an attractive, sustainable source of high-value cosmeceutical ingredients. Future pharmacological studies in biologically relevant skin models, together with comprehensive cytotoxicity, safety, formulation, and stability assessments, will be required to establish the efficacy and suitability of Artochalcone for topical cosmetic applications.

## Figures and Tables

**Table 1:** Tabular assignments of the ^13^C and ^1^H chemical shifts in compound A (Artochalcone).

Group	^13^C (ppm)	^1^H (ppm)
**1**	112.5	
2 *	165.0	
**3**	102.3	6.25
4 *	165.0	
**5**	107.7	6.31
**6**	132.3	7.63
**7**	204.4	
**8**	39.4	3.08
**9**	27.5	2.87
**10**	130.9	
**11**	119.6	6.53
**12**	112.2	6.58
**13**	142.9	
**14**	143.1	
**15**	126.6	
**16**	24.7	3.39
**17**	123.7	5.13
**18**	133.9	
**19**	39.4	1.94
**20**	26.3	2.03
**21**	123.9	5.03
**22**	130.7	
**23**	24.4	1.59
**24**	16.3	1.52
**25**	15.0	1.72

* These two resonances are overlapped in the ^13^C{^1^H} 1D spectrum.

**Table 2:** Anti-tyrosinase and antioxidant activities of the methanolic breadfruit flower extract and isolated compound, Artochalcone.

	Tyrosinase		DPPH		Nitric Oxide	
Compound	IC_50_ (μM)	IC_50_ (μg mL^−1^)	IC_50_ (μM)	IC_50_ (μg mL^−1^)	IC_50_ (μM)	IC_50_ (μg mL^−1^)
Flower extract	-	9.6	-	30.1	-	17.5
Artochalcone	28.4	11.6	20.4	8.2	20.7	8.2
Arbutin ^a^	471.5	128.4	-	-	-	-
Quercetin ^b^	-	-	7.5	2.3	-	-
Ascorbic acid ^c^	-	-	-	-	2500	440

^a,b,c^ Positive controls.

**Table 3:** Comparative protein–ligand interactions of arbutin, non-prenylated Artochalcone, and prenylated Artochalcone in the tyrosinase active site. Where multiple distances are reported, the interaction includes multiple ligand atoms. Hydrogen bond distances are from hydrogen to the acceptor atom.

Ligand	Interaction Type	Residue	Distance (Å)
Arbutin	Hydrogen bond	ASN260	2.78
	Hydrophobic	VAL283	3.74, 3.67
	π-π stacking (parallel)	HIS263	4.52
Non-prenylated Artochalcone	Hydrogen bond	ASN260	2.09
	Hydrogen bond	MET280	2.32
	Hydrogen bond	GLY281	2.45
	Hydrophobic	HIS263	3.73
	Hydrophobic	PHE264	3.67
	Hydrophobic	VAL283	3.62
	π-π stacking (parallel)	HIS263	4.09
Artochalcone	Hydrogen bond	HIS244	1.97
	Hydrogen bond	ASN260	2.41
	Hydrogen bond	SER282	2.83
	Hydrophobic	PHE264	3.57
	Hydrophobic	VAL283	3.53, 3.33, 3.65
	Hydrophobic	ALA286	3.61
	π-π stacking (parallel)	HIS263	3.92

## Data Availability

The data underlying this study are available in the published article and its supporting information, available online.

## Supplementary Materials

The following supporting information can be downloaded at https://www.mdpi.com/article/10.3390/molecules31162752/s1, Figure S1: 1H NMR of Artochalcone (CD3OD, 298 K); Figure S2: 13C NMR of Artochalcone (CD3OD, 298 K); Figure S3: 1H COSY NMR of Artochalcone (CD3OD, 298 K); Figure S4: 1H TOCSY NMR of Artochalcone (80 ms mixing time, CD3OD, 298 K); Figure S5: 1H-13C HSQC NMR of Artochalcone (CD3OD, 298 K); Figure S6: 1H-13C HMBC NMR of Artochalcone (optimized for 8 Hz coupling, CD3OD, 298 K).
