# Supplementary material for: Artochalcone—A Geranylated Dihydrochalcone from Male Inflorescences of Artocarpus altilis (Breadfruit) with Potent Anti-Tyrosinase Activity
Source: Molecules. 2026 Aug 7;31(16):2752. doi: 10.3390/molecules31162752 (PMC13515359; doi:10.3390/molecules31162752)
Supplement: Supplementary file 1 [file molecules-31-02752-s001.zip › molecules-4445372-supplementary.pdf]

SUPPLEMENTAL INFORMATION

Artochalcone – a geranylated dihydrochalcone from male inflorescences of *Artocarpus altilis* (Breadfruit) with potent anti-tyrosinase activity

Kenna L. Whitnell[1], Jackson J. Villemaire-McCutcheon[1], Ulli K.C. Bodnar[1], M. Sameer Al-Abdul-Wahid[2] and Tariq A. Akhtar[1]

[1] Department of Molecular and Cellular Biology, University of Guelph, Guelph, Ontario, N1G 2W1, Canada.
[2] NMR Centre, University of Guelph, Guelph, Ontario, N1G 2W1, Canada.

TABLE OF CONTENTS

Figure S1. 1H NMR of Artochalcone (CD3OD, 298 K).

Figure S2. 13C NMR of Artochalcone (CD3OD, 298 K).

Figure S3. 1H COSY NMR of Artochalcone (CD3OD, 298 K).

Figure S4. 1H TOCSY NMR of Artochalcone (80 ms mixing time, CD3OD, 298 K).

Figure S5. 1H-13C HSQC NMR of Artochalcone (CD3OD, 298 K).

Figure S6. 1H-13C HMBC NMR of Artochalcone (optimized for 8 Hz coupling, CD3OD, 298 K)

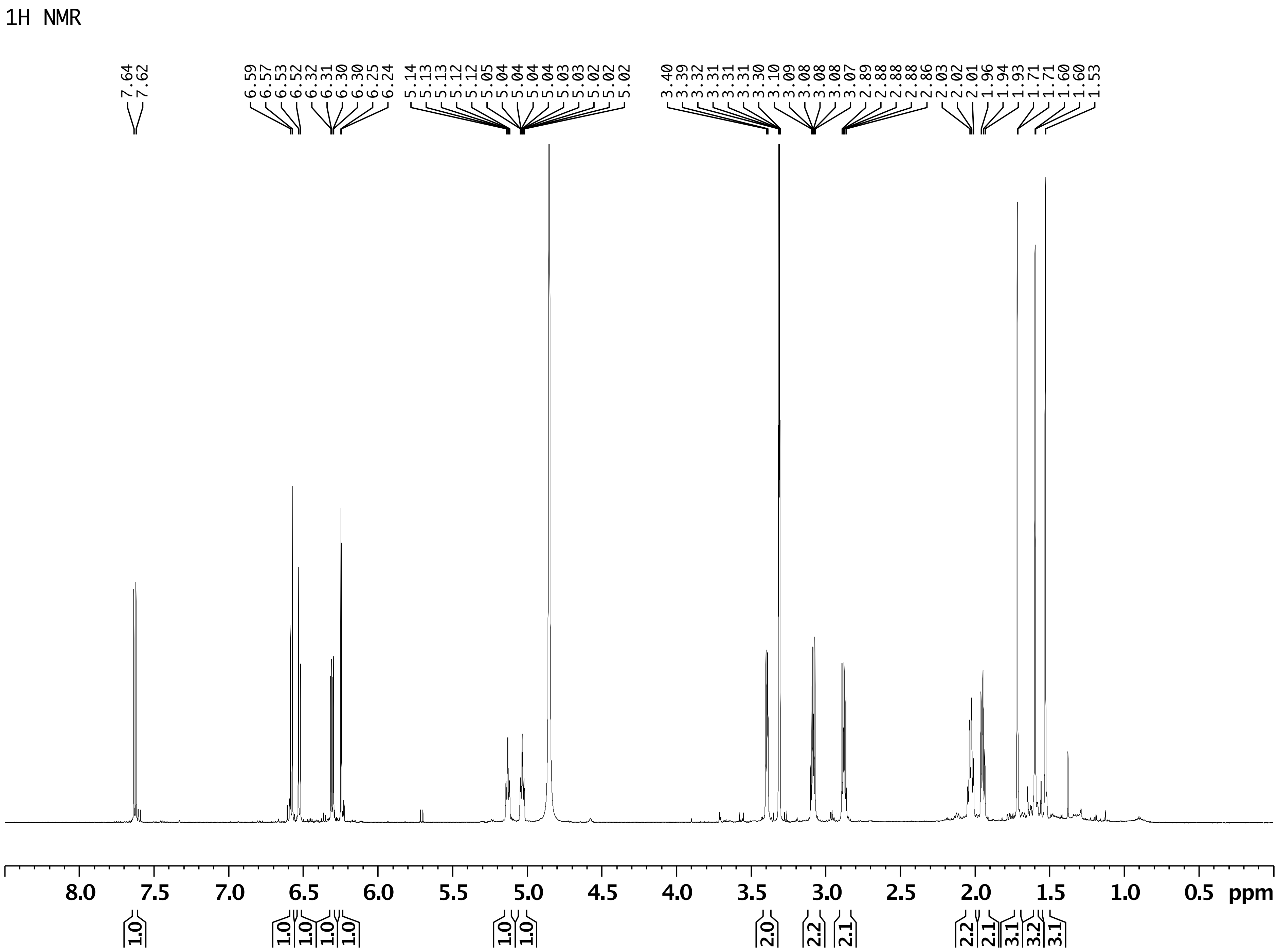

Figure S1. ^1H NMR of Artochalcone (CD_3OD, 298 K).

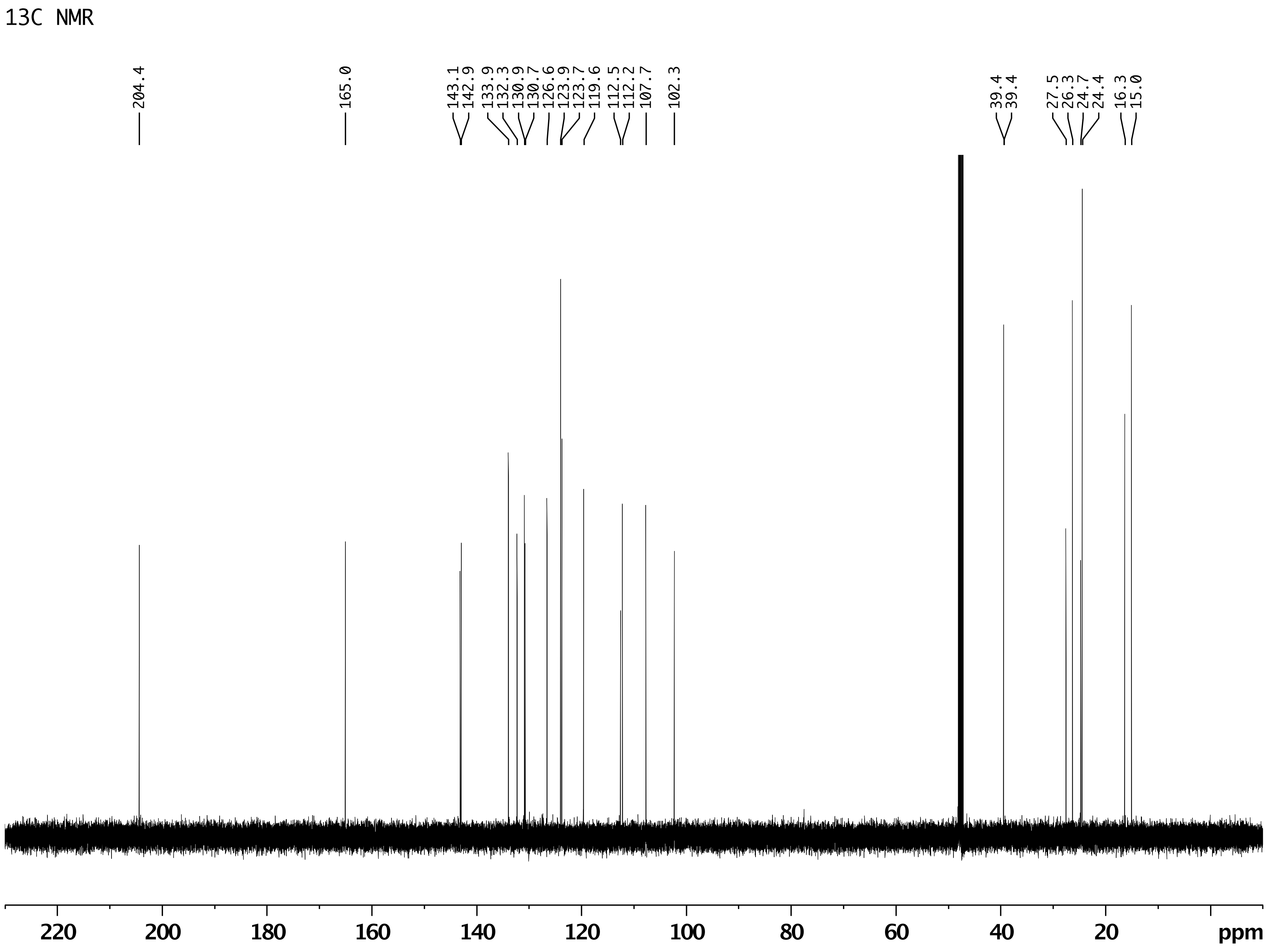

Figure S2. ^{13}C NMR of Artochalcone (CD_3OD, 298 K).

1H COSY NMR

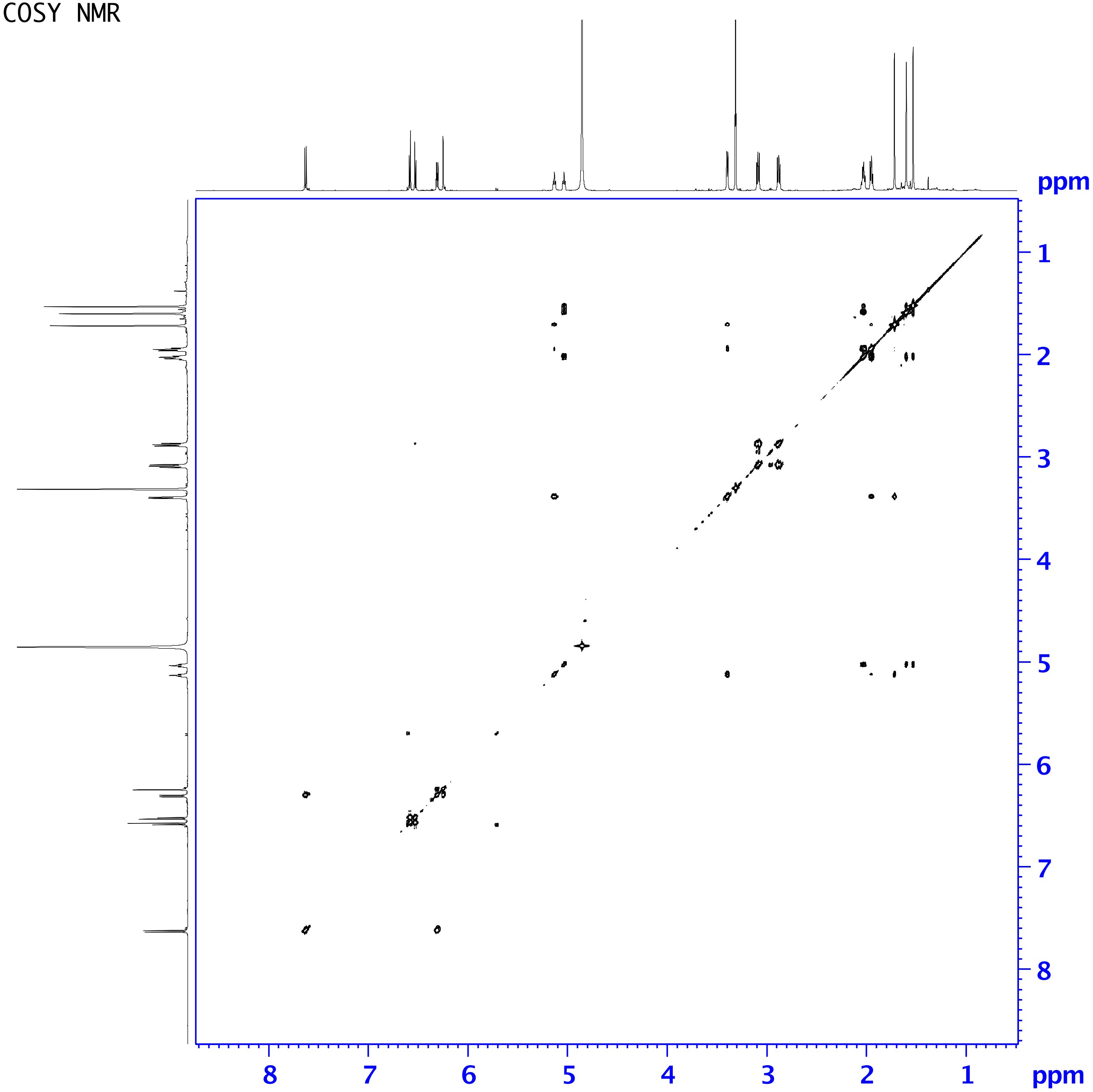

Figure S3. ^{1}H COSY NMR of Artochalcone (CD_3OD, 298 K).

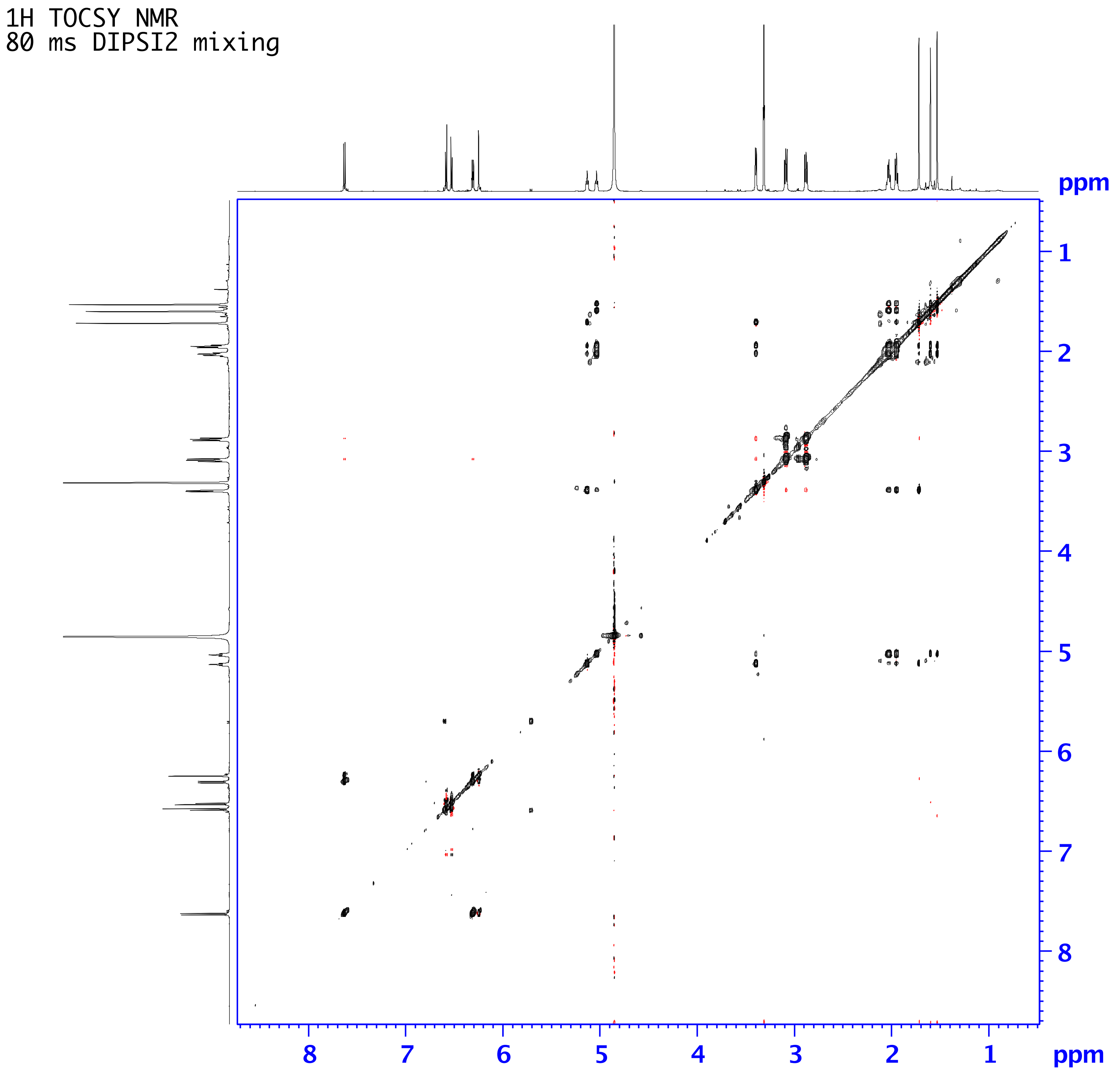

Figure S4. ^{1}H TOCSY NMR of Artochalcone (80 ms mixing time, CD_3OD, 298 K).

1H-13C HSQC NMR

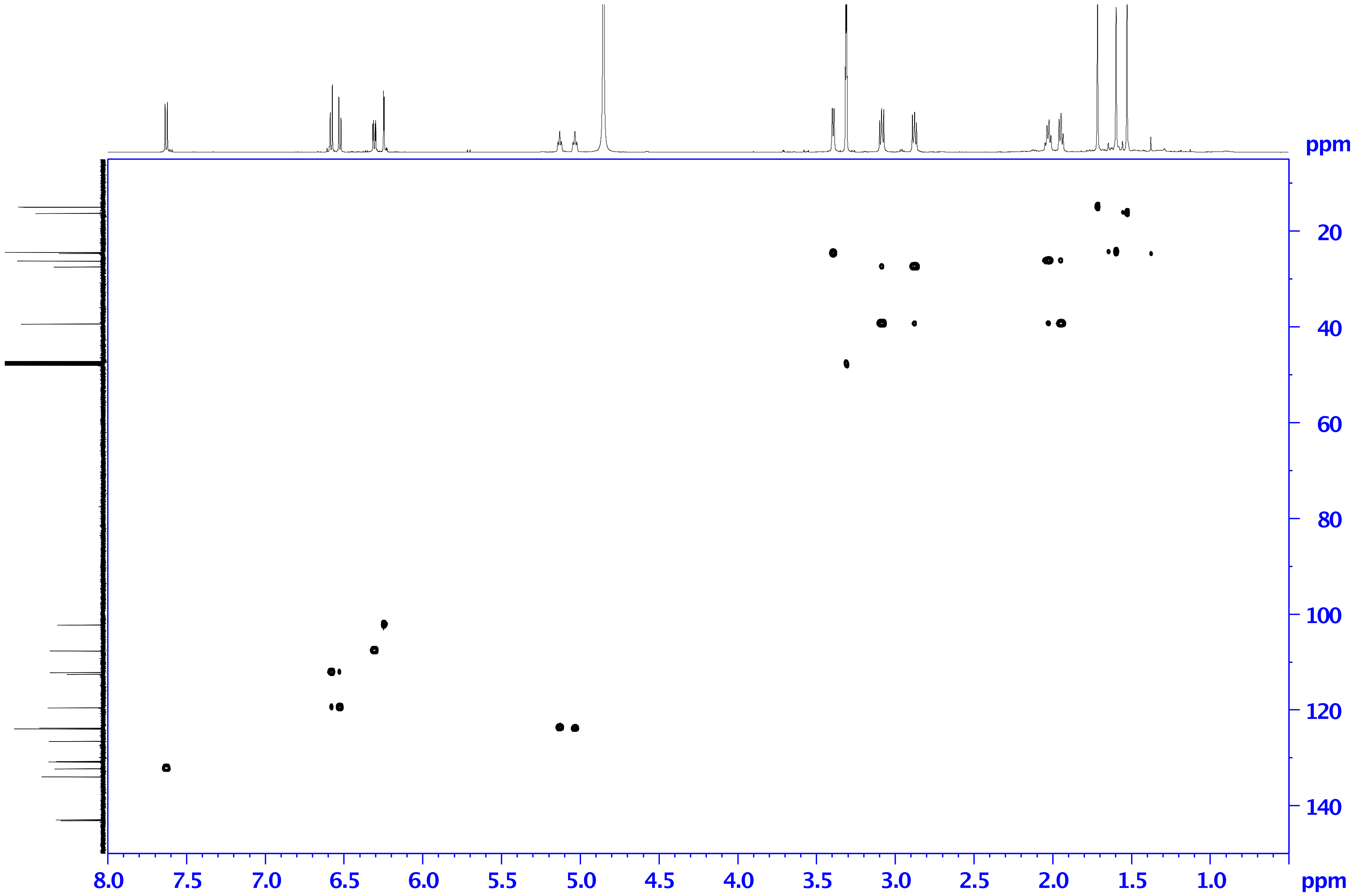

Figure S5. ^{1}H-^{13}C HSQC NMR of Artochalcone (CD_3OD, 298 K).

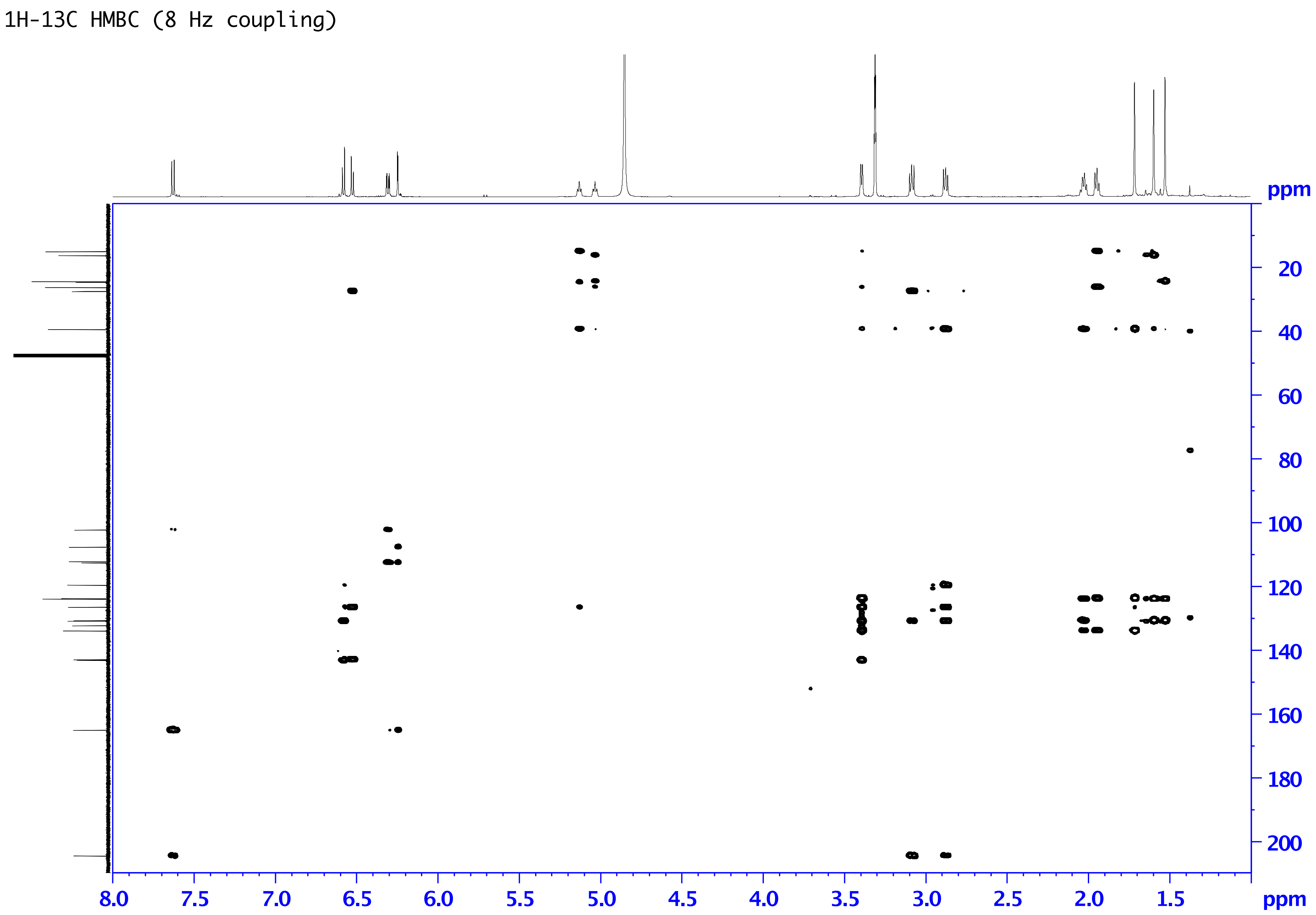

Figure S6. ^{1}H-^{13}C HMBC NMR of Artochalcone (optimized for 8 Hz coupling, CD_3OD, 298 K)
